# Interactions between ibuprofen, ACE2, renin‐angiotensin system, and spike protein in the lung. Implications for COVID‐19

**DOI:** 10.1002/ctm2.371

**Published:** 2021-04-04

**Authors:** Rita Valenzuela, Maria A. Pedrosa, Pablo Garrido‐Gil, Carmen M. Labandeira, Gemma Navarro, Rafael Franco, Ana I. Rodriguez‐Perez, Jose L. Labandeira‐Garcia

**Affiliations:** ^1^ Research Center for Molecular Medicine and Chronic diseases (CIMUS), IDIS University of Santiago de Compostela Santiago de Compostela Spain; ^2^ Networking Research Center on Neurodegenerative Diseases (CIBERNED) Madrid Spain; ^3^ Hospital Alvaro Cunqueiro University Hospital Complex Vigo Spain; ^4^ Department of Biochemistry and Physiology, Faculty of Pharmacy University of Barcelona Barcelona Spain; ^5^ Department of Biochemistry and Molecular Biology, Faculty of Biology University of Barcelona Barcelona Spain

To the Editor:

The safety of NSAIDs, particularly ibuprofen, in COVID‐19 has been openly questioned.^1^ Many review or opinion articles have discussed whether NSAIDs increase or decrease tissue levels (particularly lung levels) of ACE2 (angiotensin converting enzyme 2) and whether this may increase or decrease the severity of COVID‐19. An increase in ACE2 activity is essential to balance the tissue renin‐angiotensin system (RAS) against the inflammatory response (see Figure S1), and several recent studies have emphasized the pivotal role of tissue RAS in severity of COVID‐19.^2^ Adversely, upregulation of ACE2, as viral entry receptor, may increase cell infection. However, other proteases such as ADAM17 (TACE, TNF‐α‐converting enzyme) and transmembrane protease serine 2 (TMPRSS2) are also major mechanisms for the viral entry.^3^, ^4^ Available experimental data are scarce and controversial.

We investigated the effects of ibuprofen on lung levels of ACE2 and other major compounds of the lung RAS, both in healthy adult rats and in a well‐known rat model of metabolic syndrome (MetS; rats with obesity, hypertension, hyperglycemia). It is known that patients with MetS are more vulnerable to severe COVID‐19. In vitro, we used human alveolar type‐II pneumocyte cells to study the effects of ibuprofen on changes induced by viral spike protein on ACE2 levels, on levels of spike protein internalization, ADAM17 and TMPRSS2 activities, and on the release of major COVID‐19‐related cytokines. Details on methods are provided as Supporting Information.

In healthy adult rats, treatment with ibuprofen (40 mg/kg) significantly increased lung ACE2 expression and enzymatic activity, and increased the expression of anti‐inflammatory RAS axis receptors (Mas and angiotensin type 2; AT2); furthermore, ibuprofen induced a significant decrease in proinflammatory AT1 receptor expression (Figure 1A–C). MetS rats showed downregulation of RAS anti‐inflammatory components (ACE2, AT2 receptors, and angiotensin 1–7; Ang1–7) and increased expression of components of the RAS proinflammatory axis (AT1 receptors and AngII), which indicates the unbalance toward the proinflammatory RAS. However, treatment with ibuprofen shifted the balance toward the anti‐inflammatory RAS arm, increasing ACE2, Ang1–7, AT2, and Mas receptor levels, and decreasing AngII and AT1 receptor levels (Figure 1D–G).

**FIGURE 1:**
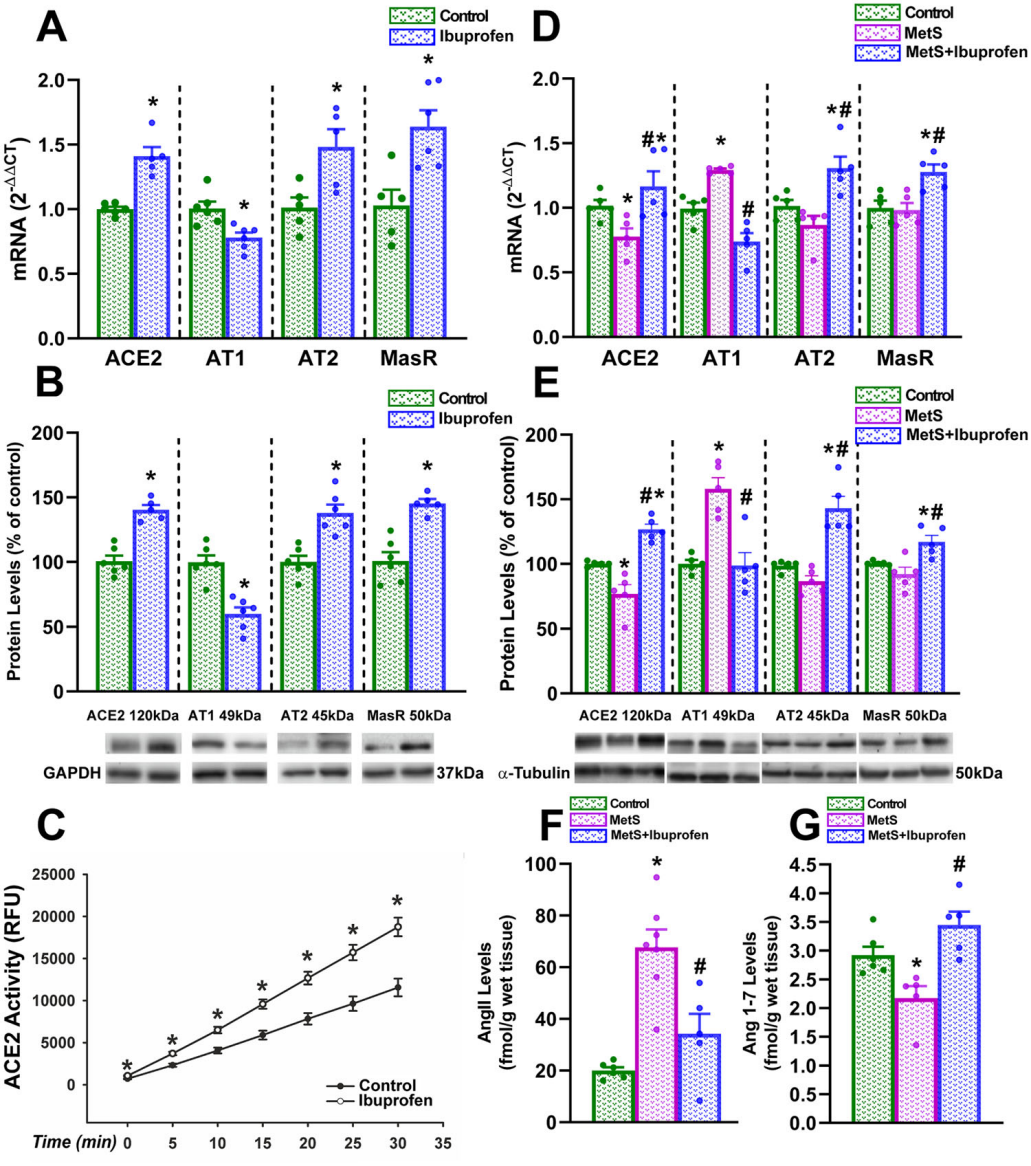
Effects of ibuprofen on mRNA (A,D) and protein (B,E) expression of ACE2 and AT1, AT2 and Mas receptors, ACE2 enzymatic activity (C), and levels of AngII (F) and Ang1–7 (G) in the lung of healthy adult rats (A–C) and rat models of metabolic syndrome (MetS; D–G). Data are mean ± SEMs. *p < .05 Relative to control adult rats; #*p* < .05 relative to untreated rat models of metabolic syndrome. For two groups comparisons Student's test or Mann–Whitney rank sum test were used. One‐way analysis of variance (ANOVA) with Student–Newman–Keuls method post hoc test or Kruskal–Wallis one‐way ANOVA on ranks with Student–Newman–Keuls method post hoc test were used for multiple comparisons

Treatment of cultures of human alveolar type‐II pneumocytes with ibuprofen also upregulated ACE2 expression 24 and 48 h after treatment (Figure 2A). Treatment of cultures with SARS‐CoV‐2 spike‐RBD led to a marked decrease in cellular levels of the transmembrane ACE2 protein (full length, 120 kDa) and cellular ACE2 activity, which was blocked by pretreatment of cultures with ibuprofen. However, treatment with spike protein increased the levels of a short ACE2 isoform (60 kDa) in cells (apparently an internalized glycosylated ACE2 polypeptide), which was also inhibited by pretreatment ibuprofen. Similarly, levels of soluble ACE2 (105 kDa) in culture medium were significantly increased by treatment of cultures with spike protein and significantly reduced by pretreatment with ibuprofen (Figure 2B–E).

**FIGURE 2:**
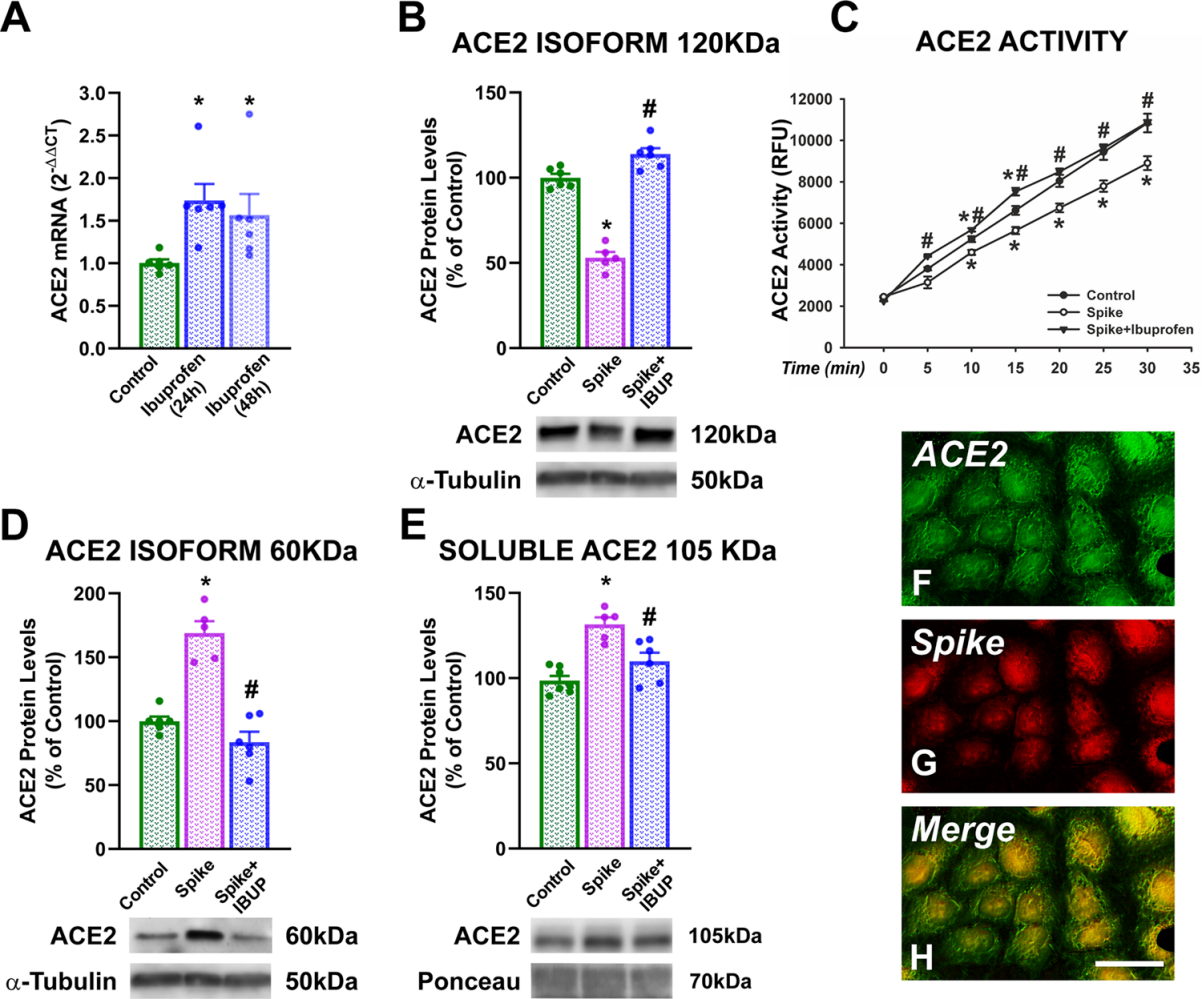
Cultures of human alveolar type‐II A549 pneumocytes. Effects of 24 or 48 h treatment with ibuprofen on cellular ACE2 mRNA expression (A). Levels of ACE2 protein (B,D,E) and enzymatic activity (C) in cells (B–D) and culture medium (E) of cultures treated with spike protein alone or spike protein and ibuprofen. Laser confocal microphotographs that show fluorescent labeling for ACE2 (green, F), spike protein (red, G), and colocalization of ACE2 and spike (H, yellow) in pneumocytes treated with spike protein. *p < .05 Relative to untreated controls; #*p* < .05 relative to cells treated with spike alone (one‐way analysis of variance [ANOVA] with Student–Newman–Keuls method post hoc test or Kruskal–Wallis one‐way ANOVA on ranks with Student–Newman–Keuls method post hoc test). Scale bar (for F–H): 25 μm

Effects of ibuprofen on spike protein internalization were studied using a laser confocal microscope. We initially observed that pneumocytes expressed ACE2 in our cultures and that spike protein was internalized by ACE2‐expressing cells (Figure 2F–H). Then, the effect of ibuprofen on SARS‐CoV‐2 spike RBD‐Fc internalization rate was measured as cytoplasmic fluorescence intensity of spike protein (Figure 3A–J). As changes in levels of internalized spike protein in different cells could be a consequence of different levels of ACE2 receptor expression in cultured A549 pneumocytes, we used ACE2‐GFP transiently transfected A549 pneumocytes to minimize possible differences between cells, and values of intracellular spike levels were expressed relative to ACE2 levels (Figure 3J). Cells in cultures treated with spike protein showed high levels of spike protein internalization (Figure 3J,D–F) relative to controls not treated with spike (Figure 3J,A–C); however, intracellular levels of spike protein were significantly reduced (around 50%) by pretreatment of cultures with ibuprofen (Figure 3J,G–I). Consistent with the above‐mentioned data, amounts of COVID‐19‐related proinflammatory cytokines (TNF‐α, IL‐6, CCL2) were significantly increased in the culture medium of pneumocytes treated with spike protein, and were significantly reduced in cultures pretreated with ibuprofen (Figure 3K–M).

**FIGURE 3:**
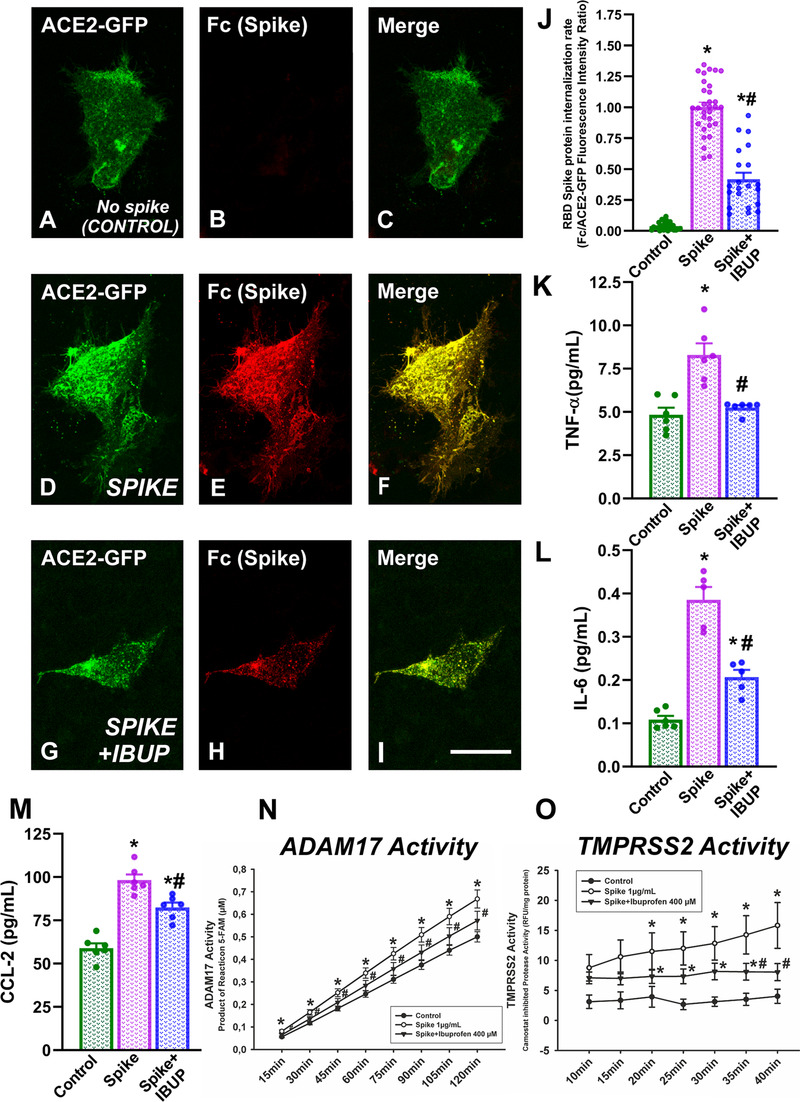
Cultures of human alveolar type‐II A549 pneumocytes. Effects of ibuprofen on the level of SARS‐CoV‐2 spike RBD‐Fc internalization quantified as cell fluorescence intensity using a laser confocal microscope (A–J). Fluorescent labeling for ACE2 (green), spike protein (red), and colocalization of ACE2 and spike (yellow) in cells not treated with spike (A–C), cells treated with spike protein only (D–F), and cells treated with spike protein and ibuprofen (G–I). Levels of TNF‐α (K), IL‐6 (L), CCL‐2 (M) in human alveolar type‐II A549 pneumocytes treated with spike protein only or spike protein and ibuprofen. ADAM17 (N) and TMPRSS2 (O) enzymatic activities in human alveolar type‐II A549 pneumocytes treated with spike protein alone or spike protein together with ibuprofen.*p < .05 Relative to untreated controls; #*p* < .05 relative to cells treated with spike alone (one‐way analysis of variance [ANOVA] with Student–Newman–Keuls method post hoc test or Kruskal–Wallis one‐way ANOVA on ranks with Student–Newman–Keuls method post hoc test). Scale bar (for all photographs): 25 μm

As several of the above‐mentioned observations, particularly ibuprofen‐induced decrease in spike protein internalization, cannot be explained by the ibuprofen‐induced increase in ACE2 expression, we investigated possible additional effects of ibuprofen on other major mechanisms involved in SARS‐CoV/spike protein internalization. First, we analyzed possible changes in ADAM17 activity because we observed that in the culture medium, pretreatment with ibuprofen inhibited the increase in levels of soluble (105 kDa) ACE2 induced by spike protein (see above). Consistent with this, we observed that ADAM17 activity was significantly upregulated in pneumocytes treated with spike protein, as compared with untreated control pneumocytes. However, this upregulation was significantly reduced by pretreatment of cultures with ibuprofen (Figure 3N). Second, we observed that TRPRSS2 activity significantly increased in pneumocytes treated with spike protein in comparison with untreated control pneumocytes, and TMPRSS2 activity was significantly reduced by pretreatment with ibuprofen (Figure 3O).

The major mechanism of action of ibuprofen is inhibition of COX‐2, and interactions between COX‐2 and RAS have previously been observed in other tissues.^5^ We observed that ibuprofen promoted the RAS anti‐inflammatory axis (Ang1–7/Mas, AT2) and inhibited the RAS proinflammatory axis (AngII/AT1/NADPH‐oxidase), which may increase ACE2 levels^6^, ^7^ and decrease ADAM17 activity^8^ by several mechanisms. Conversely, it is known that spike protein increases ADAM17 activity, resulting in ACE2 shedding, decrease in transmembrane ACE2, increase in soluble ACE2, and increase in SARSCoV/spike internalization.^3^, ^9^ Consistently, ADAM17 inhibitors were proposed as antiviral drugs.^3^ Our observation of ibuprofen‐induced decrease in TMPRSS2 activity is also supported by previous findings in other tissues.^10^

In conclusion, in the present models, ibuprofen upregulated ACE2 expression and anti‐inflammatory RAS activity in the lung. It is known that this inhibits inflammatory, fibrotic, and thrombotic responses. In human lung cell cultures, ibuprofen‐induced upregulation of the SARS‐COV‐2 main receptor (ACE2) was counteracted by ibuprofen‐induced mechanisms that reduce SARS‐COV‐2 spike protein internalization, particularly by inhibition of ADAM17 and TMPRSS2 activities (Figure S2). Although the results were obtained in rat and culture models, they support clinical studies suggesting that ibuprofen and other NSAIDs have no detrimental effects and possibly positive effects in COVID‐19 outcome.

## FUNDING INFORMATION

Axencia Galega de Innovación, Grant Number: IN845D 2020/20; Spanish Ministry of Economy and Competitiveness, Grant Numbers: RTI2018‐098830‐B‐I00 and RTI2018‐094204‐B‐I00; Spanish Ministry of Health, Grant Numbers: PI17/00828, RD16/0011/0016, and CIBERNED; Galician Government, Grant Numbers: XUGA, ED431C 2018/10, ED431G/05; FEDER (Regional European Development Fund)

## CONFLICT OF INTEREST

The authors declare that there is no conflict of interest.

## ETHICS STATEMENT

Animals were handled in accordance with the Directive 2010/63/EU, European Council Directive 86/609/EEC, and the Spanish legislation (RD53/2013). The corresponding committee at the University of Santiago de Compostela (15005/15/002) approved all animal experiments, which were performed in the Experimental Biomedicine Center (CEBEGA; University of Santiago de Compostela).

## AUTHOR CONTRIBUTIONS

Jose L. Labandeira‐Garcia, Ana I. Rodriguez‐Perez, and Rita Valenzuela designed research and experiments. Ana I. Rodriguez‐Perez, Pablo Garrido‐Gil, and Maria A. Pedrosa performed in vivo experiments. Rita Valenzuela, Maria A. Pedrosa, Rafael Franco, and Gemma Navarro performed in vitro experiments. Ana I. Rodriguez‐Perez, Rita Valenzuela, Carmen M. Labandeira, Rafael Franco, and Jose L. Labandeira‐Garcia contributed to writing, review, and critique. All the authors edited the manuscript.

## DATA AVAILABILITY STATEMENT

Data are available from the corresponding author upon reasonable request.

## Supporting information

Supporting Information

Figures S1‐S2
