# Supplementary material for: Interactions between ibuprofen, ACE2, renin‐angiotensin system, and spike protein in the lung. Implications for COVID‐19
Source: Clin Transl Med. 2021 Apr 4;11(4):e371. doi: 10.1002/ctm2.371 (PMC8019581; doi:10.1002/ctm2.371)
Supplement: Supplementary file 1 — Supporting Information [file CTM2-11-e371-s001.docx]

**MATERIALS AND METHODS**

**1. Experimental design**

**Animal models**

As the effect of NSAIDs on lung ACE2 expression is controversial, we first used rat models to investigate in vivo regulation of lung ACE2 by the NSAID ibuprofen. RT-PCR and HPLC were used to analyze the effects of ibuprofen on ACE2 and major components of lung RAS (i.e. AngII, Ang 1-7, AT1, AT2 and Mas receptors). In addition, we confirmed protein expression of ACE2 and AT1, AT2, and Mas receptors using western blot (WB), and analyzed ACE2 activity levels in lung tissue from untreated and ibuprofen-treated animals. In a first series of experiments, healthy adult rats (10 weeks old; n= 6 per group/treatment) were used. In a second series of experiments, rat models of metabolic syndrome (MetS; n= 5 per group/treatment) showing hyperglycemia, obesity, and hypertension were used. It is known that patients with metabolic syndrome are more vulnerable to severe COVID-19 outcome. Rats in the MetS group received high fat diet (60% calories fat supplemented with 4% of NaCl; D18042603, Research Diets Inc.; New Jersey, USA) for 18 weeks. To confirm the development of the MetS model we periodically measured serum triglycerides (TG), cholesterol and Glucose in rats using commercially colorimetric enzymatic kits (1001093; 1001314; 1001191; Spinreact; Girona, Spain), according to the manufacturer’s instructions. In addition, blood pressure was measured using a non-invasive pressure system MRBP (IITC Life Science, California, USA). Rats from different groups received oral treatment with the NSAID ibuprofen (40mg/Kg; I4883, Merck KGaA; Darmstadt, Germany) for 3 weeks. The powered drug was administered orally mixed with “Nocilla” hazelnut cream (Nutrexpa; Barcelona, Spain). Animals in control groups were given “Nocilla” hazelnut cream only. Doses were stablished on the basis of preliminary experiments and previous *in vivo* studies.^1^ Rats were handled in accordance with the Directive 2010/63/EU, European Council Directive 86/609/EEC and the Spanish legislation (RD53/2013). The corresponding committee at the University of Santiago de Compostela (15005/15/002) approved all animal experiments, which were performed in the Experimental Biomedicine Centre (CEBEGA; University of Santiago de Compostela). Rats were housed at constant room temperature (RT) (21–22 °C) and 12-h light/dark cycle. Rats were euthanised with an intraperitoneal overdose (800mg/Kg) of sodium pentobarbital before extraction of lung samples.

**Cell cultures**

Cultures of the human alveolar type-II pneumocyte A549 cell line were used to investigate the effects of ibuprofen on changes induced by SARS-CoV-2 Spike RBD-Fc protein. All data were obtained from at least three independent experiments with at least n= 5. First, we analyzed the impact of ibuprofen treatment (24 or 48 h) on ACE2 mRNA expression in A549 cells in culture. Then, we analyzed the effect of treatment (3 h) with viral spike on levels of ACE2 in cellular lysates (i.e. membrane-bound or intracellular forms of ACE2) and levels ACE2 in the culture medium (i.e. soluble released ACE2) in the presence or the absence of preincubation (24 h) with ibuprofen. Next, we used confocal laser microscopy to study the effect of ibuprofen on SARS-CoV-2 Spike RBD-Fc internalization rate by quantifying cytoplasmic fluorescence intensities of spike protein. We used ACE2-GFP transiently transfected A549 cells to minimize the possibility that changes in levels of internalized spike protein could be related to differences in cell levels of ACE2 receptor. Furthermore, values of intracellular spike were expressed relative ACE2 levels. We also analysed the effect of treatment with viral spike protein on the release of major pro-inflammatory cytokines (IL-6, TNF-α and CCL-2) to the culture medium in the presence or the absence of preincubation with ibuprofen. Finally, we investigated possible effects of ibuprofen on other major components involved in SARS-CoV/spike protein internalization. First, we analyzed possible changes in ADAM17 activity. Second, we analyzed possible effects on transmembrane serine protease 2 (TMPRSS2).

The human alveolar type-II pneumocyte cell line A549 (CSC-C6236X, Creative Bioarray; New York, USA), was cultured in DMEM/F12 medium (D8437, Merck KGaA; Darmstadt, Germany) supplemented with 10% FBS (10270-106, Gibco, Thermo-Fisher Scientific; Massachusetts, USA), 2 mM L-glutamine (G-7513, Merck KGaA; Darmstadt, Germany), 100 U/ml penicillin, and 100 μg/ml Streptomycin (15140-122, Gibco; Thermo-Fisher Scientific; Massachusetts, USA). Cultures were maintained at 37 °C and 5% CO_2_ in a humidified incubator. We maintained cells until maximal confluence was reached to get high levels of cellular ACE2.^2^ Cells were treated at 37°C with the NSAID ibuprofen (400μM, I4883, Merck KGaA; Darmstadt, Germany) for 24 or 48 h, or with 1 µg/ml of SARS-CoV-2 Spike RBD-Fc protein (40592-V02H, Sino Biological; Beijing, China) for 3 hours, in the presence or absence of preincubation (24 h) with ibuprofen. Doses were stablished on the basis of our preliminary experiments and previous *in vitro* studies.^3^

**2. RNA Extraction and RT-PCR**

Total RNA from rat lung tissues or A549 cell homogenate was extracted with TRI Reagent (T9424, Merck KGaA; Darmstadt, Germany) following the manufacturer's protocol. Two µg of total RNA was reversed transcribed to complementary DNA (cDNA) with nucleoside triphosphate containing deoxyribose, random primers and Moloney murine leukaemia virus reverse transcriptase (MML-V, 200U; 28025-013, Thermo-Fisher Scientific; Massachusetts, USA). QuantStudio 3 platform (Applied Biosystems; California, USA), the EvaGreen qPCR MasterMix (Applied Biological Materials Inc.; Vancouver, Canada), and the corresponding primer sequences (see below) were used to examine the relative levels of ACE2, AT1, AT2, and Mas receptors by RT-PCR. β-Actin was amplified in parallel with the genes of interest as a housekeeping gene. The comparative cycle threshold values (cycle threshold (Ct)) method (2^−ΔΔCt^) was used to examine the relative messenger RNA (mRNA) expression. A normalized value was obtained by subtracting the Ct of β-actin from the Ct of interest (ΔCt). As it is uncommon to use ΔCt as a relative expression data due to this logarithmic characteristic, the 2^−ΔΔCt^ parameter was used to express the relative expression data. Primer sequences were as follows: for Mas receptor, forward 5′-CTTTGTGGAGAACGGGAT-3′; reverse 5′-GGAGATGTCAGCAATGGA-3′ (NM_012757.2 Rattus norvegicus Mas1 proto-oncogene, G protein-coupled receptor (Mas1), mRNA); for ACE2, forward 5´-GTGGAGGTGGATGGTCTTTCAGG-3´; reverse 5´CACCAACGATCTCCCGCTTCA-3´ (NM_001012006.1 Rattus norvegicus angiotensin I converting enzyme 2 (Ace2), mRNA); for AT1, forward 5´-TTGTCTGGATAAATCACACAACCC-3´; reverse 5´-GTTAAGGGCCATTTTGTTTTTCTGG-3´ (NM_030985.4 Rattus norvegicus angiotensin II receptor, type 1a (Agtr1a), mRNA); for AT2, forward 5´-AACATCTCGCTGAAGACCAATAG-3´; reverse 5´-AGAAGGTCAGAACATGGAAGG-3´ (NM_012494.3 Rattus norvegicus angiotensin II receptor, type 2 (Agtr2), mRNA); for human ACE2, forward 5´-TTCCATGCTAACGGACCCAGGA-3´; reverse 5´- TTTGTGCACATAAGGATCCTGAAGT-3´ (NM_021804.3 Homo sapiens angiotensin I converting enzyme 2 (ACE2), transcript variant 2, mRNA).

**3. Western blotting**

We used RIPA buffer containing PMSF (P7626, Merck KGaA; Darmstadt, Germany) and protease inhibitor cocktail (P8340, Merck KGaA; Darmstadt, Germany) to lyse rat lung tissue or A549 cells samples. We analyzed total protein using the Pierce BCA Protein Assay Kit (23225, Thermo-Fisher Scientific; Massachusetts, USA). Identical amount of protein in lysates or cell culture medium was separated on 8-10% Bis-Tris polyacrylamide gel, then transferred to nitrocellulose membranes. Primary antibodies against the extracellular domain of ACE2 (1:1000, ab108252, Abcam; Cambridge, UK), AT1 (1:200, sc-31181, Santa Cruz Biotechnology Inc; Texas, USA), AT2 (1:200; sc-9040, Santa Cruz Biotechnology Inc; Texas, USA), Mas (1:1000, AAR-013, Alomone Labs; Jerusalem, Israel) receptors were used to incubate membranes overnight at 4ºC. Membranes were also reincubated with loading controls: anti-α-tubulin (1:50.000, T5168, Merck KGaA; Darmstadt, Germany) or GAPDH (1:25.000, G9545, Merck KGaA; Darmstadt, Germany), or Ponceau stain (P7170-1L, Merck KGaA; Darmstadt, Germany). As horseradish peroxidase (HRP)-conjugated secondary antibodies we used mouse anti-rabbit IgG-HRP (1:2500, sc-2357, Santa Cruz Biotechnology Inc; Texas, USA) or rabbit anti-mouse IgG-HRP (1:5000, P0260, Dako Denmark A/S; Glostrup, Denmark). Bound antibody was visualized with an Immun-Star HRP Chemiluminescent Kit (170-5044, Bio-Rad Laboratories; Madrid, Spain) and a chemiluminescence detection system (Molecular Imager ChemiDoc XRS System, Bio-Rad; Madrid, Spain). To counteract possible variability among batches, data were expressed relative to the control values.

**4. Specifity of antibodies**

The specificity of primary ACE2, AT1, AT2 and Mas receptors antibodies used in the present study was corroborated in our laboratory by WB analysis of lysates from HEK293 cells transiently transfected with the corresponding GPCR tagged to fusion tail DDK (i.e. a C-terminal DDK epitope tag DYKDDDDK) or GFP (green fluorescent protein), or preabsorption with the corresponding synthetic peptide antigen.^4,5^

**5. Detection of AngII and Ang1-7 in rat lung**

Levels of Ang II and Ang 1-7 were analyzed in rat lungs by HPLC and enzyme immunoassay as previously described^6^ with slight modifications. Rat lungs were, initially, homogenized using a polytron in an acid (HCl)-ethanol solution with EDTA and a protease inhibitor cocktail and then, by successive steps of centrifugation, incubation at -20ºC and acidification with heptafluorobutyric acid (HFBA). Then, the supernatants were applied to solid-phase extraction columns (Sep-Pak C18, Oasis HLB 1cc, WAT094225, Waters Chromatography Europe BV; Etten-Leur, The Netherlands) and angiotensin peptides were eluted with an acid (HFBA)-methanol solution. The eluant were dried in Savant (ISS110, Thermo Scientific; Massachusetts, USA) without heat, resuspended in a 12% acetonitrile solution with 0.015% HFBA and injected into the HPLC system.

Angiotensin peptides were separated using a four-step gradient with acidified (0.1% HFBA) acetonitrile (12-32% linear gradient, 32% isocratic conditions, 32-12% linear gradient and 12% isocratic conditions) on a reverse phase column (Symmetry300 C18, Waters Chromatography Europe BV; Etten-Leur, The Netherlands). The flow rate was 0.35 mL/min, and the oven was set at 25ºC. The angiotensin fractions were monitored at 220 nm with a UV-VIS detector (SPD-20 AV, Shimadzu Corporation; Kyoto, Japan), collected in a fraction collector (FRC-10A, Shimadzu Corporation; Kyoto, Japan), and dried in Savant. The pellet was resuspended and finally the levels of AngII and Ang1-7 were determined by enzyme immunoassay (LS-F10589, LSBio; Washington, USA) according to the manufacturer’s instructions. Data were expressed as fmol per gram of wet tissue.

**6. Transfection of ACE2**

A549 human epithelial lung cells were seeded at 0.35×10^6^/well density onto 12 well plates with glass cover and maintained at 37 °C in a humidified CO_2_ incubator (5% CO_2_, 95% air). We used 2 μg of ACE2 cDNA (ACE2 tGFP-tagged, RG208442, OriGene Technologies, Inc.; Maryland, USA) and the commercial transfection reagent Turbofect (R0533, Thermo Scientific; Massachusetts, USA) to transiently transfect the cells. Forty-eight hours later, cultures were treated with different compounds, fixed and processed for laser confocal microscopy studies.

**7.** **SARS-CoV-2 Spike RBD-Fc protein Internalization assay**

ACE2-tGFP transiently transfected A549 human alveolar type-II pneumocytes were treated with 1 µg/ml of SARS-CoV-2 Spike RBD-Fc protein (40592-V02H, Sino Biological; Beijing, China) for 3 hours at 37°C in the presence or the absence of 24 h preincubation with ibuprofen. Then, cells were fixed and incubated overnight at 4 ºC with a mouse monoclonal antibody against human IgG-Fc (1:500, ab99757, Abcam; Cambridge, UK) in DPBS containing 1% BSA, 2% normal goat serum and 0.05% Triton X-100. Cells were then treated with a fluorescent secondary antibody (1:200, Alexa Fluor 568- conjugated goat anti-mouse IgG, A11004, Molecular Probes; Oregon, USA) for 2.5 h at RT. Mounting was performed with Thermo Scientific™ Shandon™ Immu-Mount (9990412, Thermo Scientific; Massachusetts, USA) and a confocal laser microscopy (AOBS-SP5X; Leica Microsystems GmbH; Wetzlar, Germany) was used to estimate the internalization rate. Sequential scan was performed to avoid any potential overlap with the LAS AF software (Leica Microsystems GmbH; Wetzlar, Germany). Around 25-30 optical fields per sample were randomly chosen using a 63x objective. RBD Spike protein internalization rate was expressed as the ratio of the fluorescence intensities measured at 568nm (human Fc signal) and 488nm (ACE2-GFP signal) excitation wavelengths. Fluorescence intensity was measured at the level of cellular cytoplasm and the background of each image was subtracted before calculating the ratio with the Software Leica LAS AF. For the analysis, we used images of cells at a unique plane and the same conditions of laser intensities/exposure times for the entire experiment.

**8. ACE2 activity** **assay**

Rat lung tissue (20 μg) or human A549 cell lysate (5μg) was used to determine ACE2 activity using a commercial ACE2 activity assay kit (AS-72086, AnaSpec; California, USA) following the manufacturer’s specifications. The kit determines ACE2 activity using the Mca/Dnp fluorescence resonance energy transfer (FRET) peptide (10 μM). The fluorescence of Mca is quenched by Dnp. Cleavage of the substrate by the enzyme produces a separation into two fragments, and the fluorescence of Mca is measured at excitation/emission = 330/390 nm with an Infinite M200 multiwell plate reader (Tecan Austria GmbH; Grödig, Austria). We used the specific ACE2 inhibitor DX600 (1 µM), included as a control in the same kit (AS-72086, AnaSpec; California, USA), to confirm specificity of the assay.

**9. ADAM17 activity assay**

ADAM17 activity was determined in human A549 cell lysate (20μg) using a commercial TACE activity assay kit (AS-72085, AnaSpec; California, USA) following the manufacturer’s specifications. The kit uses the 5-FAM/QXL® 520 based fluorescence resonance energy transfer (FRET) peptide (1 μM) and the fluorescence of 5-FAM is quenched by QXL® 520. A cleavage of the substrate by the enzyme produces a separation into two fragments and the fluorescence of 5-FAM is measured at excitation/emission = 490/520 nm using an Infinite M200 multiwell plate reader (Tecan Austria GmbH; Grödig, Austria). We used the TACE inhibitor TAPI-0 (10 μM), included as a control in the same kit to confirm specificity of the assay.

**10. TRPRSS2 activity assay**

Protease activity of human A549 cells lysate was measured using a commercial Protease Assay kit (AS-71124, AnaSpec; California, USA) following the manufacturer’s specifications. The kit uses casein labeled with HiLyte Fluor 488. Proteolytic cleavage of this quenched casein-HiLyte Fluor 488 conjugate yields brightly green fluorescence, which was continuously monitored at excitation/emission= 488/520 nm using an Infinite M200 multiwell plate reader (Tecan Austria GmbH; Grödig, Austria). We measured total protease activity of 125μg of human A549 cells lysate in 20mM sodium phosphate buffer pH 8, and calculated TMPRRS2 activity using the specific TMPRRS2 inhibitor Camostat mesylate (100μM, SML0057, Merck KGaA; Darmstadt, Germany).^7^

**11. Enzyme immunoassays (EIA)**

The effect of treatments on release of pro-inflammatory markers was analysed in A549 cell cultures. Culture supernatants were collected and centrifuged at 2,000g for 10 min to eliminate cell’s debris. Levels of pro-inflammatory chemokine CCL-2 (MPC-1) (ab179886, Abcam; Cambridge, UK) and pro-inflammatory cytokines IL-6 (BMS213HS, Thermo-Fisher Scientific; Massachusetts, USA) and TNF-α (BMS223HS, Thermo-Fisher Scientific; Massachusetts, USA) were determined in the culture medium using commercially available specific EIA kits according to the manufacturer’s instructions. Finally, the concentration of the corresponding cytokine was quantified using a specific standard curve (4PL curve fit).

**12. Statistical analysis**

All statistics were performed with the aid of Graphpad Prism 8 and SigmaPlot 11.0 (Systat Software, Inc.; California, USA). Datasets were tested for normality with the Kolmogorov–Smirnov test. If the dataset passed the normality test, parametric tests were used: Student’s t test were used for two group comparisons and one-way ANOVA followed by the Student-Newman-Keuls Method for multiple comparisons. For non-parametric data, two group comparisons were carried out by Mann-Whitney Rank Sum Test and multiple comparisons by Kruskal-Wallis One Way Analysis of Variance on Ranks test followed by Student-Newman-Keuls Method or Dunn's Method were used. Differences were considered statistically significant at p < 0.05.

**REFERENCES**

1. Qiao W, Wang C, Chen B, et al. Ibuprofen attenuates cardiac fibrosis in streptozotocin-induced diabetic rats. *Cardiology.* 2015;131:97-106.

2. Gandhi CK, Holmes R, Gewolb IH, Uhal BD. Degradation of Lung Protective Angiotensin Converting Enzyme-2 by Meconium in Human Alveolar Epithelial Cells: A Potential Pathogenic Mechanism in Meconium Aspiration Syndrome*. Lung.* 2019;197:227-233.

3. Endo H, Yano M, Okumura Y, Kido H. Ibuprofen enhances the anticancer activity of cisplatin in lung cancer cells by inhibiting the heat shock protein 70. *Cell Death Dis.* 2014;5:e1027.

4. Labandeira-Garcia JL, Valenzuela R, Costa-Besada MA, Villar-Cheda B, Rodriguez-Perez AI. The intracellular renin-angiotensin system: Friend or foe. Some light from the dopaminergic neurons. *Prog Neurobiol.* 2020:101919.

5. Valenzuela R, Costa-Besada MA, Iglesias-Gonzalez J, et al. Mitochondrial angiotensin receptors in dopaminergic neurons. Role in cell protection and aging-related vulnerability to neurodegeneration. *Cell Death Dis.* 2016;7:e2427

6. Brosnihan KB, Chappell MC. Measurement of Angiotensin Peptides: HPLC-RIA. *Methods Mol Biol.* 2017;1527:81-99

7. Cheng YW, Chao TL, Li CL, et al. Furin Inhibitors Block SARS-CoV-2 Spike Protein Cleavage to Suppress Virus Production and Cytopathic Effects. *Cell Rep.* 2020; 33:108254.
