# Supplementary material for: Interactions between ibuprofen, ACE2, renin‐angiotensin system, and spike protein in the lung. Implications for COVID‐19
Source: Clin Transl Med. 2021 Apr 4;11(4):e371. doi: 10.1002/ctm2.371 (PMC8019581; doi:10.1002/ctm2.371)
Supplement: Supplementary file 2 — Figures S1‐S2 [file CTM2-11-e371-s002.pdf]

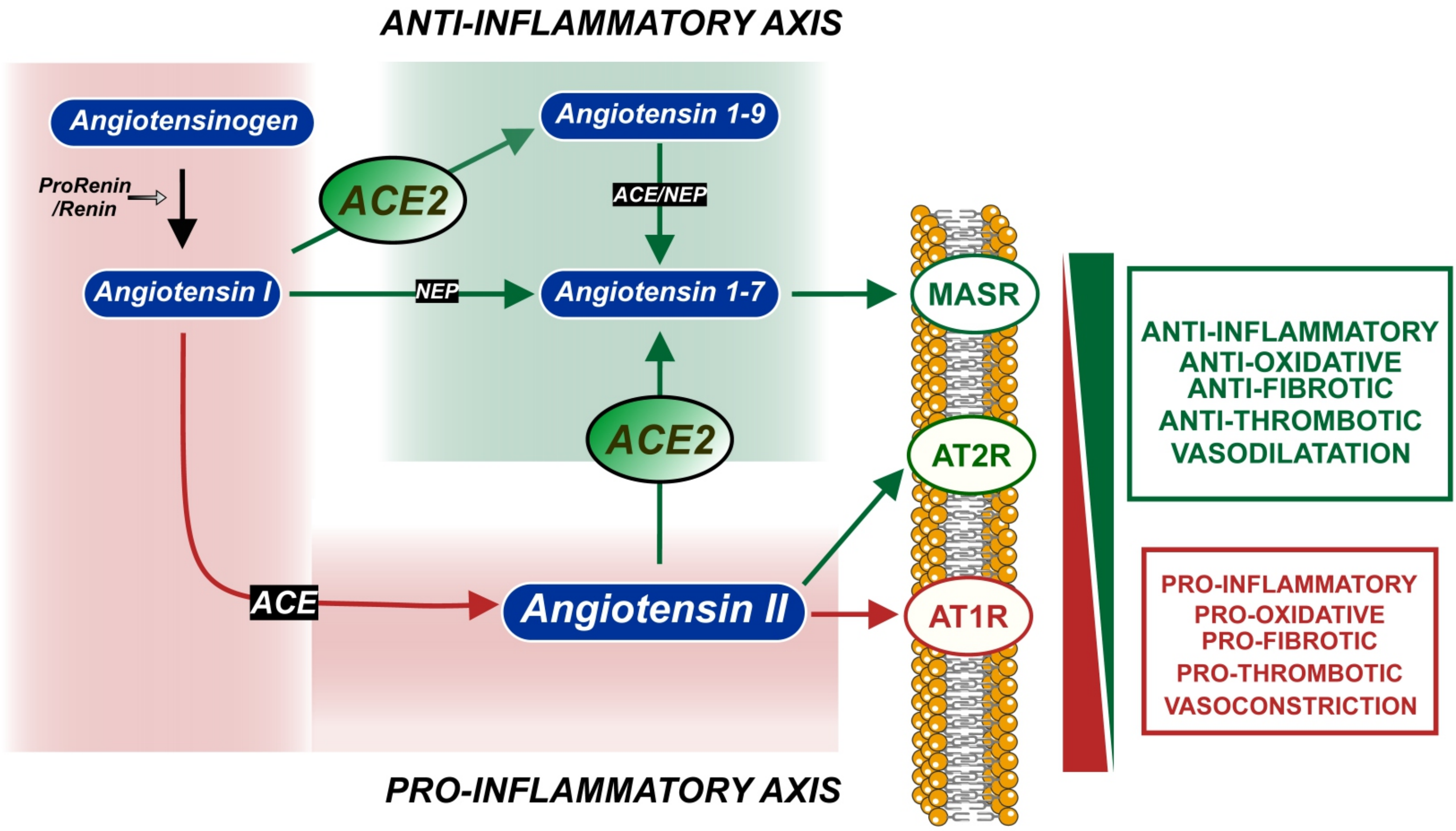

SUPPLEMENTARY FIGURE 1

The renin–angiotensin system (RAS) is constituted by two opposite arms that must be perfectly balanced. First, a RAS oxidative pro-inflammatory arm/axis (in red) mainly constituted by angiotensin II (the most effective RAS peptide) and its type I receptor (AT1R). Second, an anti-oxidative anti-inflammatory arm/axis (in green) constituted by Angiotensin II/AT2R, and particularly Angiotensin 1-7 /Mas receptors (MASR). Angiotensin II is synthetized by two enzymes, prorenin/renin and angiotensin converting enzyme (ACE), sequentially acting on the precursor protein angiotensinogen and angiotensin I, respectively. Angiotensin converting enzyme 2 (ACE2) is a key component for the RAS balance, as ACE2 (with the aid of additional peptidases such as Neprilysin, NEP) converts peptides of the pro-inflammatory arm (i.e. Angiotensin I and particularly Angiotensin II) into peptides of the anti-inflammatory arm (i.e. Angiotensin 1-9, and particularly Angiotensin 1-7).

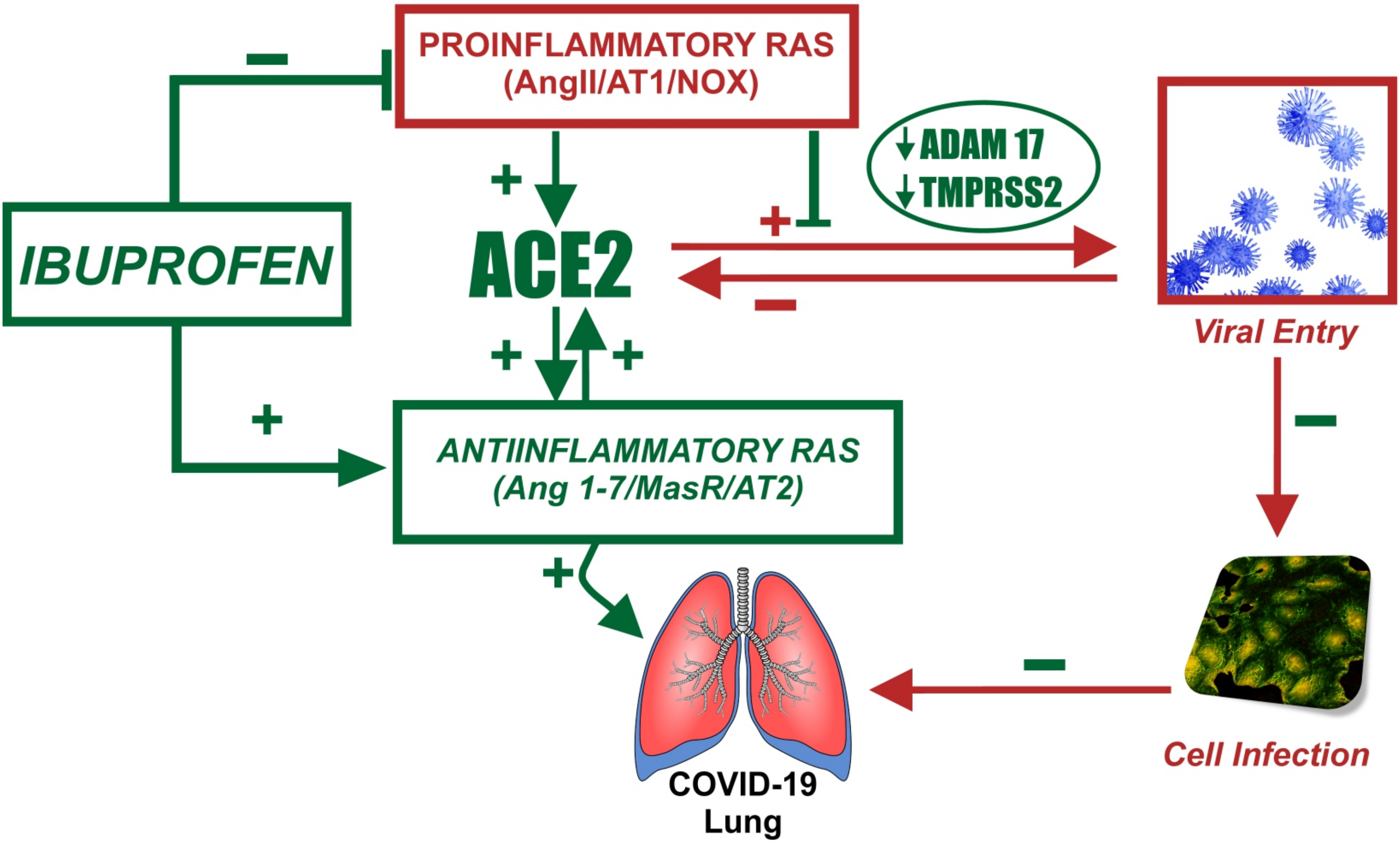

SUPPLEMENTARY FIGURE 2

Ibuprofen inhibits the pro-inflammatory RAS axis and enhances anti-inflammatory RAS arm activity leading to an increase in ACE2 levels. The increase in ACE2 activity further enhances the anti-inflammatory axis. This leads to anti-inflammatory, anti-fibrotic and anti-thrombotic effects in the lung. The ibuprofen-induced upregulation of ACE2 leads to an increase in levels of viral receptors, which may enhance cell infection and reduce levels of transmembrane ACE2. However, ibuprofen, via RAS modulation and possibly additional mechanisms, also inhibits ADAM17 and TMPRSS2 activities, which reduces viral entry and cell infection. Green lines, beneficial effects; red lines, detrimental effects.
